# Thyrotoxicosis With Medullary Thyroid Carcinoma: A Rare Endocrine Diagnostic Challenge

**DOI:** 10.7759/cureus.78096

**Published:** 2025-01-27

**Authors:** G Balamurugan, N Selvaraj, Sahana Kannan

**Affiliations:** 1 Department of Surgical Oncology, Coimbatore Medical College Hospital, Coimbatore, IND

**Keywords:** elevated calcitonin levels, elevated carcinoembryonic antigen (cea), hyperthyroidism, medullary thyroid carcinoma, thyroidectomy, thyrotoxicosis

## Abstract

The incidence of a hyper-functioning thyroid gland presenting alongside an underlying malignancy rarely occurs. Herein, we present a rare case of a 56-year-old female patient presenting with features of hyperthyroidism only to be later diagnosed with an underlying malignancy, medullary thyroid carcinoma (MTC). Ultrasonography (USG) of the neck revealed a suspicious nodule with microcalcifications in the right lobe of the thyroid. Subsequent CT imaging of the neck indicated possible cervical and mediastinal lymph node involvement. Fine-needle aspiration cytology (FNAC) confirmed the presence of high-grade thyroid carcinoma. The patient underwent total thyroidectomy with neck node dissection after which her symptoms subsided. She was well at her last follow-up.

## Introduction

Thyrotoxicosis occurs due to elevated levels of thyroid hormones, which can adversely affect various tissues and can stem from a variety of causes. It often presents with symptoms such as palpitations, shortness of breath, increased appetite, sweating, and intolerance to heat. It is characterized by elevated levels of free T3 and T4 as well as suppressed TSH levels due to negative feedback [1]. Hyperthyroidism can result from conditions such as Graves' disease, overactive thyroid nodules, or thyroiditis [2]. Risk factors for developing thyrotoxicosis include a family history of thyroid disease, recent pregnancy or delivery, autoimmune diseases like type one diabetes, pernicious anemia, and Addison's disease as well as female gender and age over 60 [3].

The diagnosis of thyroid carcinoma is rarely considered when thyrotoxicosis is present. The association is not evident in most patients because the suppressed TSH levels inhibit the growth of TSH-dependent malignant nodules, and the hyperfunctioning (hot) nodules overshadow the typically non-functioning (cold) malignant ones in imaging and clinical evaluation [4]. Thyroid carcinoma is one of the most common endocrine malignancies. Thyroid cancer is more common in females than in males, with an incidence rate of around 3.9% in females and 1.3% in males [5]. There are four different types of thyroid cancers; among these, medullary thyroid carcinoma (MTC) is the rarest type, accounting for only 3-4% of all thyroid cancers [6]. MTC is a tumor that arises from the parafollicular or C cells of the thyroid gland and produces excessive amounts of calcitonin, causing a massive increase in its serum levels. It is considered to be aggressive with 70% of cases having cervical lymphadenopathy. Distant metastasis may be present in about 10% of patients at presentation with the common sites being the lung, liver, bone, and brain which leads to a very poor prognosis. It has a variable and unpredictable course [7]. Approximately 75% of cases are sporadic and the rest are familial, being a part of MEN 2A or MEN 2B syndrome [8]. 

Patients with thyroid carcinoma will usually be in a euthyroid or hypothyroid state. The incidence of thyroid cancer in hyperthyroid patients varies from 0.1 to 21% [9]. The possibility of hidden malignancy in patients presenting with symptoms of hyperthyroidism mustn't be overlooked as it may eventually lead to distant metastasis and death. In this report, we share a case of thyrotoxicosis with underlying malignancy, its clinical presentation, and management.

## Case presentation

A 56-year-old female patient presented to the surgical oncology department with a one-year history of swelling in the front of the neck. The swelling was insidious in onset and had progressively increased in size without associated pain. Six months after first noticing the swelling, she began to experience symptoms including excessive fatigue, weight loss, heat intolerance, excessive sweating, insomnia, and intermittent episodes of loose stools (three episodes per day). After six months of symptoms, she sought medical attention. She has no family history of thyroid disorders.

Physical examination revealed nodular enlargement of the right lobe of the thyroid gland which appeared to be 10 x 6 cm in size and multiple palpable lymph nodes in the upper, middle, lower jugular, and supraclavicular regions. No eye signs were present and a review of all other systems was normal. Vitals were within normal ranges. Thyroid function tests revealed elevated levels of free T3 and T4 and a suppressed TSH level. Chemiluminescent immunoassay showed elevated levels of calcitonin and carcinoembryonic antigen (Table 1).

**Table 1:** Thyroid function tests and chemiluminescent assay FT3: free triiodothyronine; FT4: free tetraiodothyronine; TSH: thyroid-stimulating hormone; CEA: carcinoembryonic antigen; N/A: not applicable for CEA and calcitonin as they remain unaffected by the administration of antithyroid drugs.

Investigation	Patient Value	After two weeks of antithyroid medication	Normal Range
FT3	5.50 pg/ml	2.90 pg/ml	2.0-4.40 pg/ml
FT4	2.20 ng/dl	0.99 ng/dl	0.93-1.70 ng/dl
TSH	0.023 uIU/ml	1.2 uIU/ml	0.4-4.5 uIU/ml
Calcitonin	97.5 pg/ml	N/A	<5 pg/ml
CEA	39.99 ng/ml	N/A	<2.5 ng/ml

**Figure 1:**
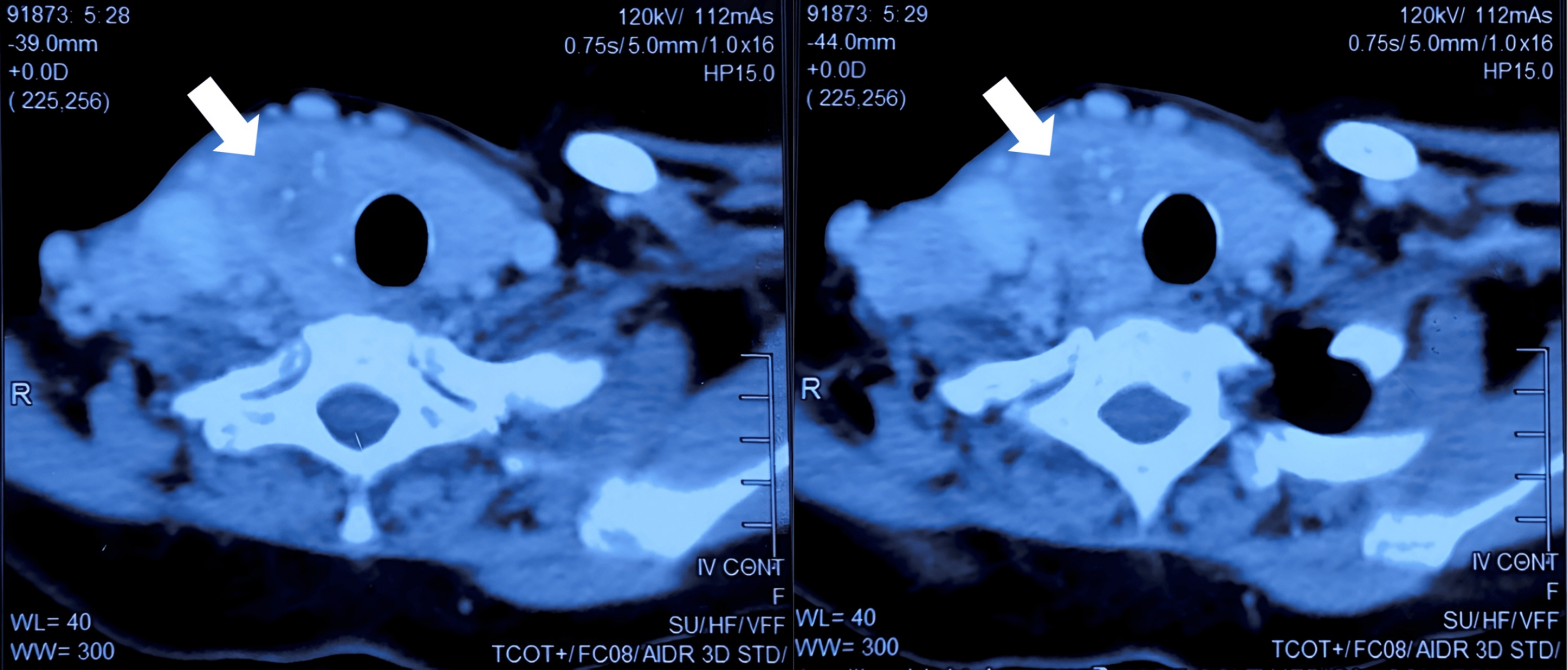
Neck CT at presentation showing axial sections of the tumor with calcifications as indicated by the arrows

She was initially diagnosed with hyperthyroidism based on clinical examination as well as laboratory findings and was prescribed anti-thyroid medications to attain a euthyroid state. The patient was subjected to further investigations like USG and CT of the neck to detect the presence of malignancy and evaluate cervical and mediastinal nodal metastasis. USG of the neck revealed a heterogeneous and enlarged thyroid gland. Heterogeneous echotexture was noted with increased vascularity in the left lobe and isthmus of the thyroid gland and a solid lesion of size 4.2 x 2.7 cm was seen in the right lobe. Enlarged lymph nodes were noted in the right second, third, and fourth levels of the neck with the largest one measuring 2.5 x 2.7 cm in level four. CT scan revealed a heterogeneously enhancing nodule of size 4.1 x 5.1 x 4.1 cm in the right lobe with multiple tiny calcific foci observed within it. A few enlarged lymph nodes were seen on the right side of the neck at levels four and five with tiny calcifications noted within them, with the largest measuring 3.9 x 2.8 cm (Figure 1).

**Figure 2:**
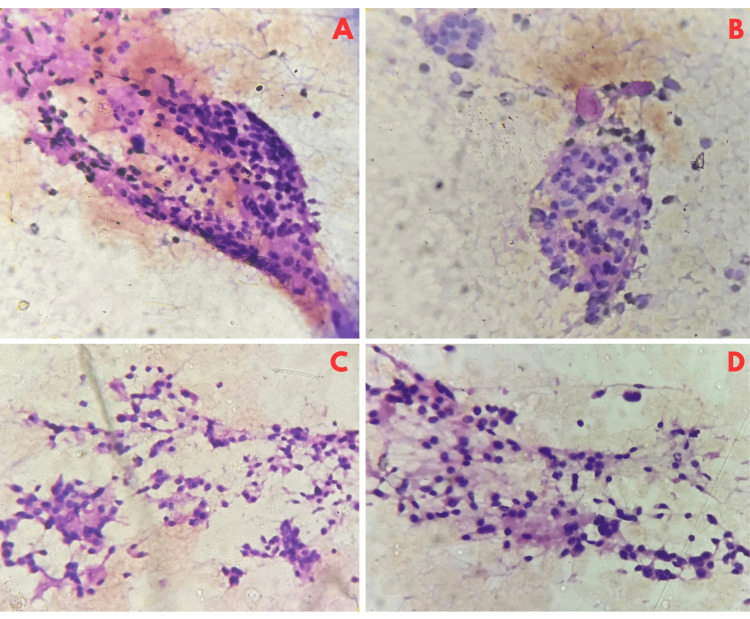
Cytological findings (A) Cohesive clusters of cells with uniform nuclei which show rounding and overlapping which is suspicious of malignancy, H&E stain, magnification x40; (B) Cluster of cells arranged in a cohesive group with individual cells showing hyperchromatic nuclei with granular chromatin and inconspicuous nucleoli as well as anisonucleosis indicative of dysplasia, H&E stain, magnification x40; (C) Dispersed and loosely cohesive clusters of cells, H&E, magnification x40; (D) Cells of varying sizes and shapes, dispersed nuclei and some clustering with scant colloid in the background depicting a neoplastic process, H&E, magnification x40.

After two weeks of taking anti-thyroid medication, fine-needle aspiration cytology (FNAC) was performed for confirmation of imaging findings. FNAC showed features suggestive of Bethesda category six, possibly high-grade thyroid carcinoma (Figure 2).

**Figure 3:**
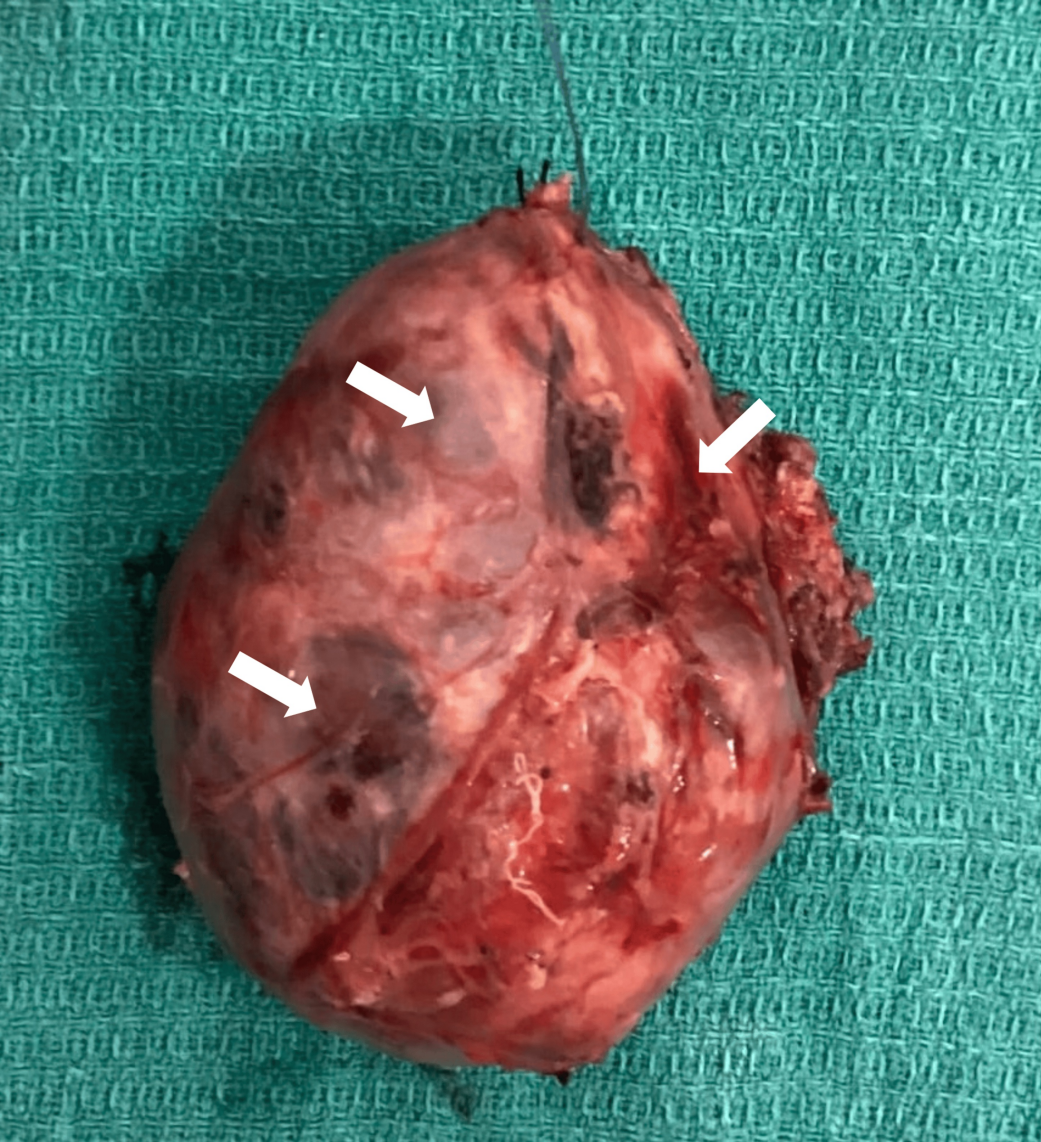
Gross examination of the thyroid gland post total thyroidectomy showing areas of cysts, necrosis and hemorrhage as indicated by the arrows

After carrying out imaging and cytological studies, the patient was diagnosed with thyrotoxicosis and high-grade thyroid carcinoma. Surgery was done two days later after ensuring that the patient was in a euthyroid state to prevent the occurrence of a thyroid storm intraoperatively. Total thyroidectomy with central compartment neck node dissection and right functional neck dissection was performed. Gross examination of the thyroid gland revealed findings as shown in Figure 3.

Bilateral recurrent laryngeal nerves and superior and inferior parathyroid glands were preserved. The postoperative period was uneventful apart from a minor change in voice. No symptoms of hypocalcemia were observed. Post-operative histopathological examination revealed the malignancy to be MTC. Calcitonin and carcinoembryonic antigen levels were measured post-surgery to check for residual disease in the nodes and appeared to be normal. No adjuvant therapy was given. The patient was further counseled on the importance of medication compliance and follow-up care, which included work-up for RET germline mutations and serial monitoring of baseline calcitonin and carcinoembryonic antigen (CEA) levels.

## Discussion

In cases like the present one, where thyrotoxicosis is present, clinicians may attribute neck swelling and thyroid function abnormalities to benign hyperfunctioning conditions, potentially delaying the diagnosis of an underlying malignancy. Previous case reports have similarly described the coexistence of thyrotoxicosis and thyroid carcinoma, suggesting that this overlap is rare but significant.

According to a systematic review and meta-analysis by Rehman et al., which included 22 studies spanning from 1992 to 2018, solitary thyroid nodules (as seen in our case) were found to carry a greater risk of thyroid carcinoma compared to multinodular goiter [10]. Only 14 cases of MTC associated with hyperthyroidism have been documented so far worldwide [11]. A case report by Sapalidis et al. describes a case of Grave’s disease with MTC and highlights the importance of thorough clinical evaluation, and laboratory and imaging studies so that an early diagnosis can be made and timely management can be carried out [12]. Mirfakhraee et al. reported that among 77 patients, 28 were found to have thyroid carcinoma within a solitary hyperfunctioning nodule, with papillary thyroid carcinoma (PTC) being the most common form of hyperfunctioning thyroid carcinoma [13]. MTC isn’t typically associated with an overactive thyroid gland due to its origin from C cells and not follicular cells which normally produce thyroid hormones. It offers a poor prognosis especially in people >55 years of age, with women being commonly affected [14]. It is aggressive and delayed diagnosis can lead to distant metastasis which has a 10-year survival rate of only 40% [15]. In our case, toxic adenoma with high-grade MTC was promptly managed, highlighting the importance of not neglecting the possibility of hidden malignancy despite the presence of a hyperfunctioning thyroid gland.

In patients with thyrotoxicosis, the likelihood of malignancy is reduced compared to those with a euthyroid status as thyroid growth-promoting factors like TSH are lower. However, in Graves' disease which is a major cause of thyrotoxicosis, thyroid-stimulating immunoglobulin (TSI) can still stimulate thyroid follicular epithelial cells to develop tumors [16]. Thyrotoxicosis can also occur in the setting of differentiated thyroid cancer due to functioning metastases as stated by Krishnaja et al [5].

The diagnosis of sporadic MTC can be arrived at using FNA biopsy and immunohistochemical staining for calcitonin. Preoperative diagnosis is difficult due to its rare incidence and its presentation as a solitary nodule. Hence post-operative histopathological examination as done in our case helps make an accurate diagnosis [17].

Treatment consists of medical management of thyrotoxicosis which normalizes the thyroid hormone levels followed by surgical management of the underlying malignancy. Thyrotoxicosis is managed using thionamides such as methimazole or propylthiouracil along with beta-blockers like propranolol to achieve a euthyroid state before surgery [18]. Surgical resection with total thyroidectomy is the primary treatment for MTC. A unilateral neck dissection is performed when the tumor measures less than 1 cm, whereas a bilateral neck dissection is recommended for tumors larger than 1 cm. In inherited cases, total prophylactic thyroidectomy is recommended by age five or upon mutation detection [19]. 

Postoperative management depends on postoperative serum calcitonin and CEA levels to detect residual disease. Elevated levels of calcitonin and CEA postoperatively may signify recurrence or spreading as well. Management of recurrent or residual MTC and distal metastasis includes surgical resection, external beam radiation therapy, and local therapies like radiofrequency ablation, cryoablation, and embolization. Systemic therapies such as chemotherapy, kinase inhibitors (vandetanib and cabozantinib), and immune checkpoint inhibitors are used for non-resectable and advanced cases [17]. Radioactive iodine therapy and TSH suppression therapy are used only in the case of differentiated thyroid carcinoma (papillary and follicular thyroid carcinoma) and not in MTC. The underlying rationale is that papillary and follicular thyroid carcinoma are derived from follicular cells whereas MTC is derived from parafollicular or C cells which do not take up radioactive iodine and don’t have TSH receptors [19, 20]. 

## Conclusions

Patients with thyrotoxicosis are not immune to developing thyroid carcinoma, and this paper covers a rare scenario where MTC coexists with a hyperfunctioning thyroid gland. To aid clinicians in managing such cases, it is essential to maintain a high index of suspicion for thyroid carcinoma in patients presenting with hyperthyroidism, particularly if they exhibit atypical features such as a palpable thyroid nodule, asymmetric thyroid enlargement, or lack of response to standard treatments for hyperthyroidism. Early identification through a combination of clinical, radiological, and cytological assessments is crucial for optimal management. FNAC is a vital diagnostic tool for confirming the presence of malignancy. Preoperative preparation should include the use of antithyroid drugs or beta-blockers to achieve a euthyroid state, minimizing surgical risk. Total thyroidectomy remains the cornerstone of treatment. Preoperative and postoperative monitoring of calcitonin and CEA levels is crucial. It helps in the diagnosis of MTC and aids in disease management as well as the assessment of treatment efficacy. A multidisciplinary approach is essential to formulate a treatment plan specific to the needs of the patient, improving both prognosis and quality of life. 
